# Quantitative criteria for improving performance of buccal DNA for high-throughput genetic analysis

**DOI:** 10.1186/1471-2156-13-75

**Published:** 2012-08-25

**Authors:** Jessica G Woo, Lisa J Martin, Lili Ding, W Mark Brown, Timothy D Howard, Carl D Langefeld, Charles J Moomaw, Mary Haverbusch, Guangyun Sun, Subba R Indugula, Hong Cheng, Ranjan Deka, Daniel Woo

**Affiliations:** 1 Cincinnati Children’s Hospital Medical Center, 3333 Burnet Avenue, Cincinnati, OH, 45229–3039, USA; 2 University of Cincinnati College of Medicine, P.O. Box 670525, Cincinnati, OH, 45267–0525, USA; 3 Wake Forest School of Medicine, Medical Center Blvd, Winston-Salem, NC, 27157, USA

**Keywords:** Buccal, Blood, DNA, Quality, Minor allele frequency (MAF), Genetic

## Abstract

**Background:**

DNA from buccal brush samples is being used for high-throughput analyses in a variety of applications, but the impact of sample type on genotyping success and downstream statistical analysis remains unclear. The objective of the current study was to determine laboratory predictors of genotyping failure among buccal DNA samples, and to evaluate the successfully genotyped results with respect to analytic quality control metrics. Sample and genotyping characteristics were compared between buccal and blood samples collected in the population-based Genetic and Environmental Risk Factors for Hemorrhagic Stroke (GERFHS) study (https://gerfhs.phs.wfubmc.edu/public/index.cfm).

**Results:**

Seven-hundred eight (708) buccal and 142 blood DNA samples were analyzed for laboratory-based and analysis metrics. Overall genotyping failure rates were not statistically different between buccal (11.3%) and blood (7.0%, p = 0.18) samples; however, both the Contrast Quality Control (cQC) rate and the dynamic model (DM) call rates were lower among buccal DNA samples (p < 0.0001). The ratio of double-stranded to total DNA (ds/total ratio) in the buccal samples was the only laboratory characteristic predicting sample success (p < 0.0001). A threshold of at least 34% ds/total DNA provided specificity of 98.7% with a 90.5% negative predictive value for eliminating probable failures. After genotyping, median sample call rates (99.1% vs. 99.4%, p < 0.0001) and heterozygosity rates (25.6% vs. 25.7%, p = 0.006) were lower for buccal versus blood DNA samples, respectively, but absolute differences were small. Minor allele frequency differences from HapMap were smaller for buccal than blood samples, and both sample types demonstrated tight genotyping clusters, even for rare alleles.

**Conclusions:**

We identified a buccal sample characteristic, a ratio of ds/total DNA <34%, which distinguished buccal DNA samples likely to fail high-throughput genotyping. Applying this threshold, the quality of final genotyping resulting from buccal samples is somewhat lower, but compares favorably to blood. Caution is warranted if cases and controls have different sample types, but buccal samples provide comparable results to blood samples in large-scale genotyping analyses.

## Background

Genetic studies involving high-throughput methods, such as genome-wide association studies or sequencing, are increasingly important and often rely on large cohorts of participants or patients. In the context of large genetic epidemiologic studies, DNA derived from buccal cells has the advantage of being readily mailed to remote regions whereas a blood draw frequently requires an in-person contact, and buccal collection may result in higher subject participation [1]. While newer options, such as saliva collection, offer high-quality non-invasive methods for collecting DNA [2,3], many older epidemiologic studies, as well as a variety of other applications such as forensics, rely on buccal DNA samples for genetic analysis [4,5]. The primary concern about buccal samples is that they may be more likely to fail genotyping, either due to low yield or lower or more variable quality of DNA. Several studies have explored the quantity and quality of DNA extracted from buccal samples, with mixed conclusions about their utility [3,4,6-10].

Sample and processing metrics to predict buccal DNA sample failure and improve genotyping quality are therefore essential. Additionally, no studies to our knowledge have examined the impact of sample type on estimates of allele frequency or other metrics likely to directly impact statistical analysis and interpretation of high-throughput genetic studies. Our group has previously explored the yield and utility of buccal DNA samples in the context of high-throughput SNP genotyping [11], and has shown that buccal DNA provide reasonable yields and concordance with paired blood samples. Other studies have also shown success with buccal-derived DNA in this context [12,13]. However, our previous study was limited in scope and used genotype call rates and concordance rates as the only quality control metrics.

The objective of the current study, therefore, is to determine methods for predicting which buccal DNA samples are likely to provide unacceptable genotyping results, and to evaluate the performance of successful buccal genotyping with respect to factors important for correct statistical analysis and interpretation of the resulting data. We report that buccal DNA samples with <34% double-stranded DNA (dsDNA) to total DNA were 18 times more likely to fail than samples with ≥34% dsDNA. Also, we report that buccal samples exhibited lower call rates than blood samples; but samples that passed QC had high quality genotype clustering and exhibited minor allele frequency estimates comparable to HapMap. Taken together, these results indicate some loss of statistical power but minimal statistical bias when using these samples in high-throughput genetic studies.

## Results

Comparisons of subject and sample characteristics by sample type are presented in Table 1. Buccal samples and blood samples did not differ with respect to the subject’s age (p = 0.07), gender (p = 0.9) or stroke status (p = 0.17), as expected given the matched study design and balanced sample plating design. The sample types also did not differ on subjects’ cigarette smoking status (p = 1.0), cigar smoking (p = 1.0) or frequency of alcohol use (p = 0.3; data not shown). Average daily intake of caffeinated beverages likewise did not differ by sample type (coffee: p = 0.6, tea: p = 0.08; soda: p = 0.98).

**Table 1:** Patient and quality control characteristics by sample type

**N**	**All**	**Buccal**	**Blood**
	**850**	**708 (83%)**	**142 (17%)**
**Patient**			
Age (years ± SD)	66.7 ± 14.8	67.2 ± 14.8	64.6 ± 14.9
Sex (% male)	417 (49.1%)	348 (49.2%)	69 (48.6%)
Stroke status (% cases)	433 (50.9%)	353 (49.9%)	80 (56.3%)
Cigarette smoker (% current)	163 (19.2%)	136 (19.2%)	27 (19.0%)
**Pre-genotyping QC**			
QC Failure (%)^a^	90 (10.6%)	80 (11.3%)	10 (7.0%)
cQC <0.4	75 (8.8%)	69 (9.7%)	6 (4.2%)
DM call rate <83%	65 (7.6%)	60 (8.5%)	5 (3.5%)
Gender mismatch	24 (2.8%)	19 (2.7%)	5 (3.5%)
cQC rate	2.08 ± 0.94	2.00 ± 0.94	2.53 ± 0.82***
Among passed samples	2.33 ± 0.65	2.24 ± 0.65	2.69 ± 0.55***
Among failed samples	0.05 ± 0.43	0.01 ± 0.37	0.36 ± 0.71
DM call rate	93.1 ± 7.8%	92.7 ± 8.3%	95.1 ± 4.0% ***
Among passed samples	95.3 ± 2.8%	95.1 ± 2.9%	96.0 ± 1.9% ***
Among failed samples	74.7 ± 11.7%	73.5 ± 11.7%	83.9 ± 6.9% **

N (%) or mean ± SD presented.

^a^ Failure subsets do not add to total failed samples, as samples frequently fail for more than one reason.

** p < 0.01 blood vs. buccal samples; ***p < 0.0001 blood vs. buccal samples.

Quality control metrics differed somewhat between blood and buccal samples (Table 1). Overall failure rates did not differ significantly (11.3% buccal, 7.0% blood, p = 0.18), and the proportion of samples that met any specific failure threshold also did not significantly differ by sample type (cQC failure, p = 0.06; DM call rate failure, p = 0.13; gender mismatch, p = 0.4). However, buccal samples exhibited lower average cQC rates (p < 0.0001) and DM call rates (p < 0.0001) than blood samples. Interestingly, successful buccal samples had lower cQC rates (p < 0.0001) and DM call rates (p < 0.0001) than successful blood samples. Similarly, failed buccal samples also had lower DM call rates than failed blood samples (p < 0.01), which suggests an overall downward shift in the distribution of these quality metrics among buccal samples.

### Phase I: Predicting pre-genotyping QC failure in Buccal samples

Considering all sample quality and quantity variables; time between sample collection and genotyping; and relevant patient lifestyle factors (Table 2), lower ds/total DNA ratio was significantly (p < 0.0001) associated with buccal sample failure, while higher total DNA concentration was marginally associated (p = 0.05). Failed buccal samples were also collected more recently than those that did not fail (p < 0.0001). Because higher total DNA concentration helps define ds/total DNA ratio but was less strongly associated, we limited further analysis of buccal sample failure to ds/total DNA ratio and time since sample collection.

**Table 2:** Predictors of quality control failure among buccal samples

**N**	**All buccal**	**QC passed**	**QC failed**	**P-value t-test or χ**^**2**^	**p-value Wilcoxon or Exact**
	**708**	**628**	**80**		
Years since sample collection	7.9 [5.3, 10.0]	8.0 [5.6, 10.0]	5.3 [4.2, 9.0]	<0.0001	<0.0001
Current cigarette smoker (%)	136 (19.2%)	116 (18.5%)	20 (25%)	0.16	0.18
Current cigar smoker (%)	21 (3%)	17 (2.7%)	4 (5%)	0.26	0.28
Average daily coffee intake (cups)	1.43 (1.28, 1.59)	1.39 (1.23, 1.54)	1.79 (1.16, 2.42)	0.23	0.25
Average daily tea intake (cups)	0.42 (0.33, 0.51)	0.42 (0.32, 0.51)	0.42 (0.19, 0.66)	0.96	0.77
Average daily sodas	0.75 (0.62, 0.87)	0.74 (0.60, 0.87)	0.85 (0.54, 1.16)	0.50	0.06
260/280 Ratio	1.81 (1.80-1.81)	1.81 (1.80-1.81)	1.82(1.80-1.84)	0.42	0.32
260/230 Ratio	1.17 (1.15- 1.20)	1.18 (1.15-1.21)	1.14 (1.06-1.21)	0.38	0.39
Total DNA (ng/μl)^a^	87.4 (82.3-92.8)	85.6 (80.6-91.8)	102.5 (87.4-121.5)	0.06	0.05
ds DNA (ng/μl)^a^	55.7 (52.5-59.1)	56.8 (53.5-60.3)	49.4 (41.3-59.1)	0.12	0.09
ds/total DNA ratio	0.66 (0.65-0.68)	0.68 (0.67-0.69)	0.52 (0.46-0.57)	<0.0001	<0.0001

Mean (95% confidence limits of mean) or n (%) presented; median [interquartile range] presented for years since sample collection.

^a^ Back-transformed from log values (geometric mean).

**Figure 1:**
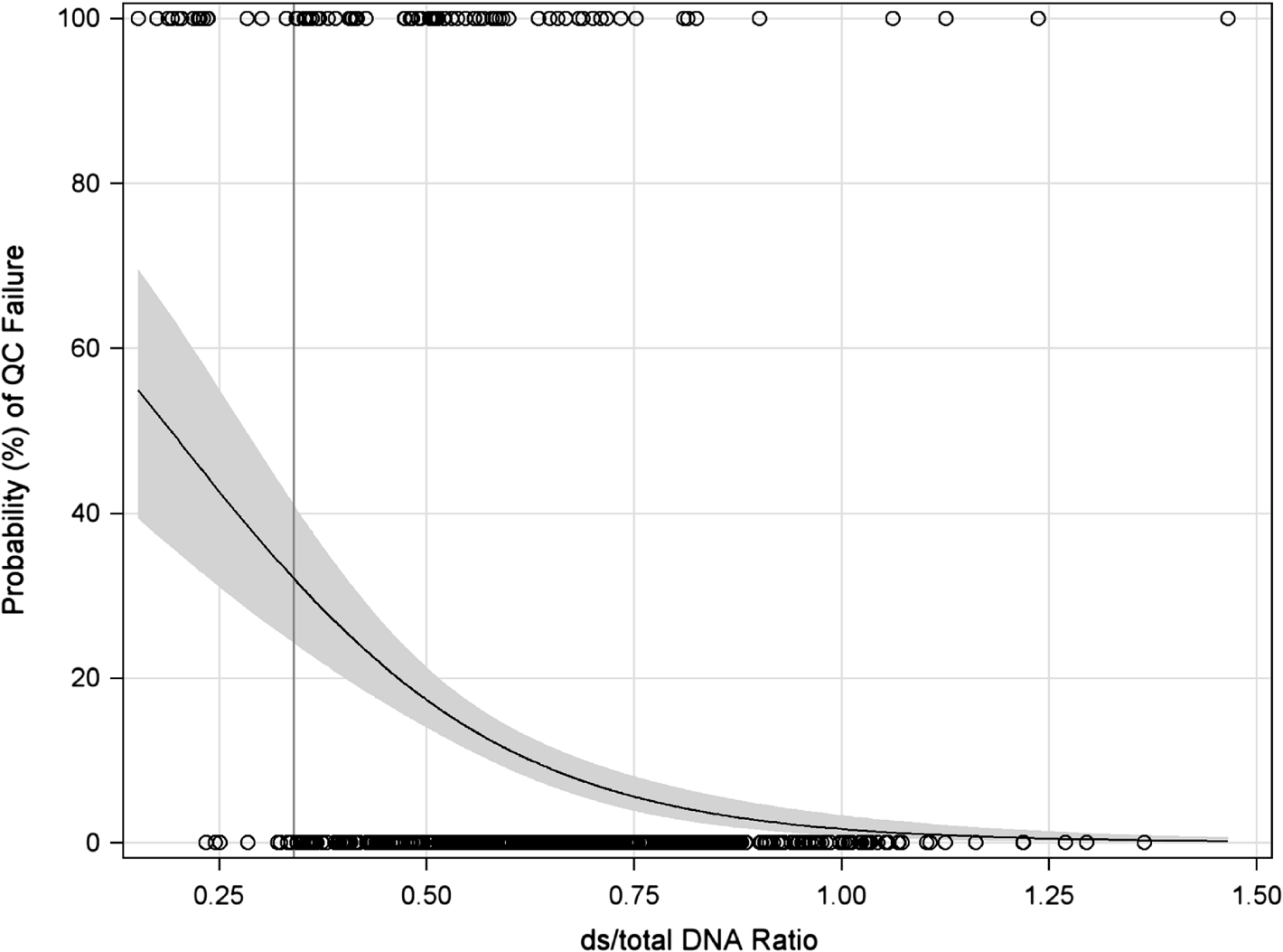
**Probability of sample failure by ds/total DNA ratio.** Predicted probability of sample pre-genotyping failure from logistic regression analysis. Line represents predicted probability and shaded area is 95% confidence limits.

From logistic regression analyses, each 10% absolute increase in ds/total DNA ratio (e.g., from 20% to 30%) is associated with a 40% lower odds of sample failure (OR = 0.61, 95% CI = 0.52–0.70, p < 0.0001), with a significant area under the ROC curve of 0.74 (95% confidence interval: 0.67–0.80, p < 0.0001). Importantly, the probability of sample failure increased significantly with very low ds/total ratios (Figure 1). Years since sample collection was significant when added to this logistic model (OR = 0.86, 95% CI = 0.77-0.95, p = 0.004), but did not materially alter the findings for ds/total DNA ratio (OR = 0.62, 95% CI = 0.55-0.72, p < 0.0001; ROC AUC = 0.75). Using this analysis, we established a threshold of ds/total ratio ≥ 0.34 during the study to determine which samples should be processed, which balanced sensitivity for failure of 20.5% with specificity for failure of 98.7% in the first 3 plates run. Overall, samples with ds/total ratio ≥ 0.34 experienced a 9.5% failure rate (63 of 662), compared with a 65% failure rate (15 of 23) among those that did not meet the ds/total DNA threshold (OR = 17.8, 95% CI: 7.3–43.7, p < 0.0001; Table 3). By contrast, the higher “optimal” threshold ds/total ratio of ≥0.58 (70.5% sensitivity and 69.7% specificity) would have reduced the QC failure rate to 5.5%, but adoption of this threshold would have resulted in the elimination of 34% of our buccal samples, 77% (180/233) of which would ultimately have passed QC.

**Table 3:** Sensitivity and specificity of ds/total DNA ratios to detect buccal sample failure

**ds/total DNA ratio threshold**	**N (%) of samples meeting threshold**	**Sensitivity**	**Specificity**	**Positive predictive value**	**Negative predictive value**
<0.34	23 (3.3%)	20.5%	98.7%	65.2%	90.5%
<0.58	223 (34.0%)	70.5%	69.7%	22.75%	94.5%

### Phase II: Buccal versus blood genotype quality

**Figure 2:**
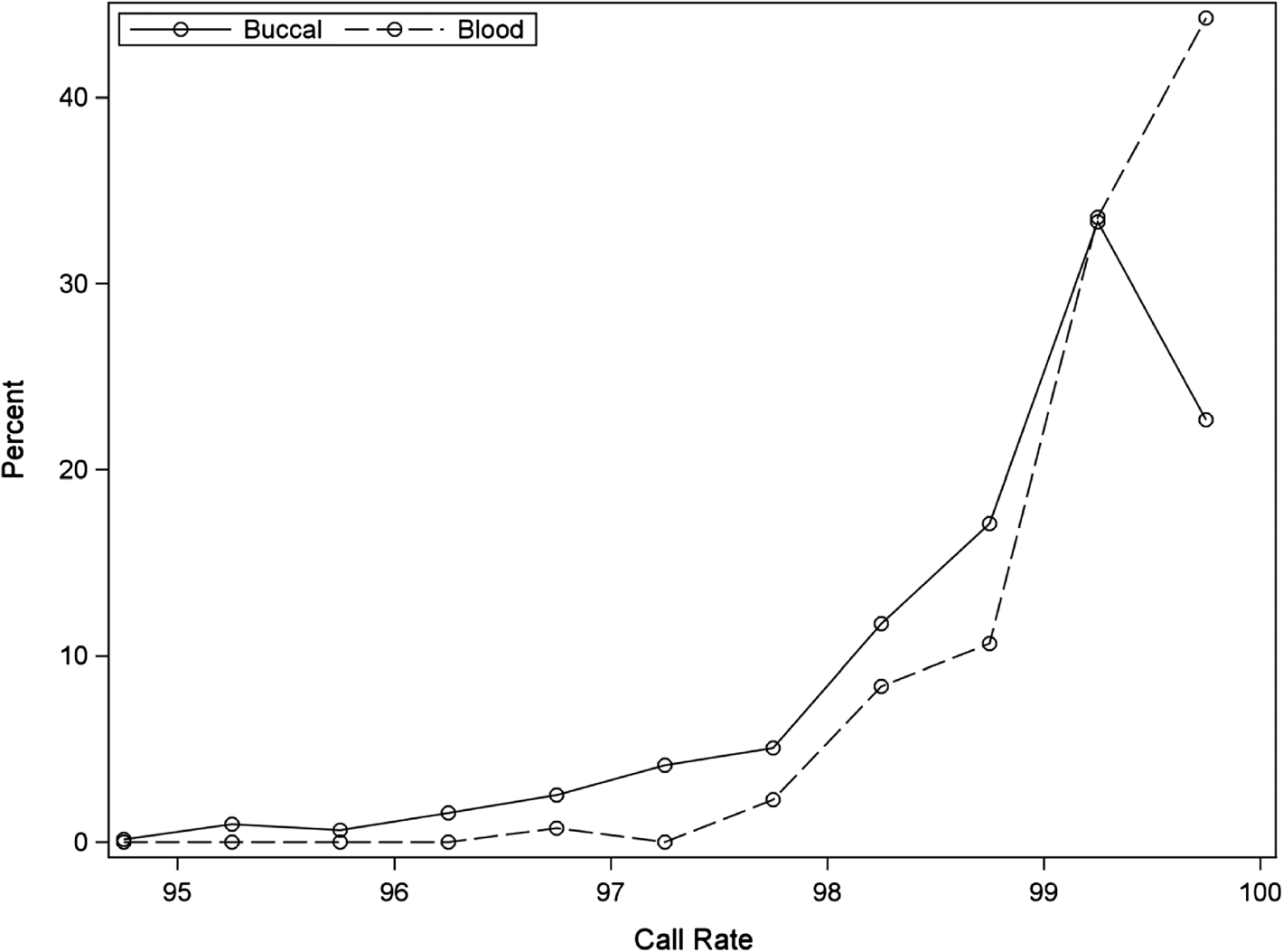
**Call rates by sample type.** Histogram and smoothed spline of sample call rates by sample type.

**Figure 3:**
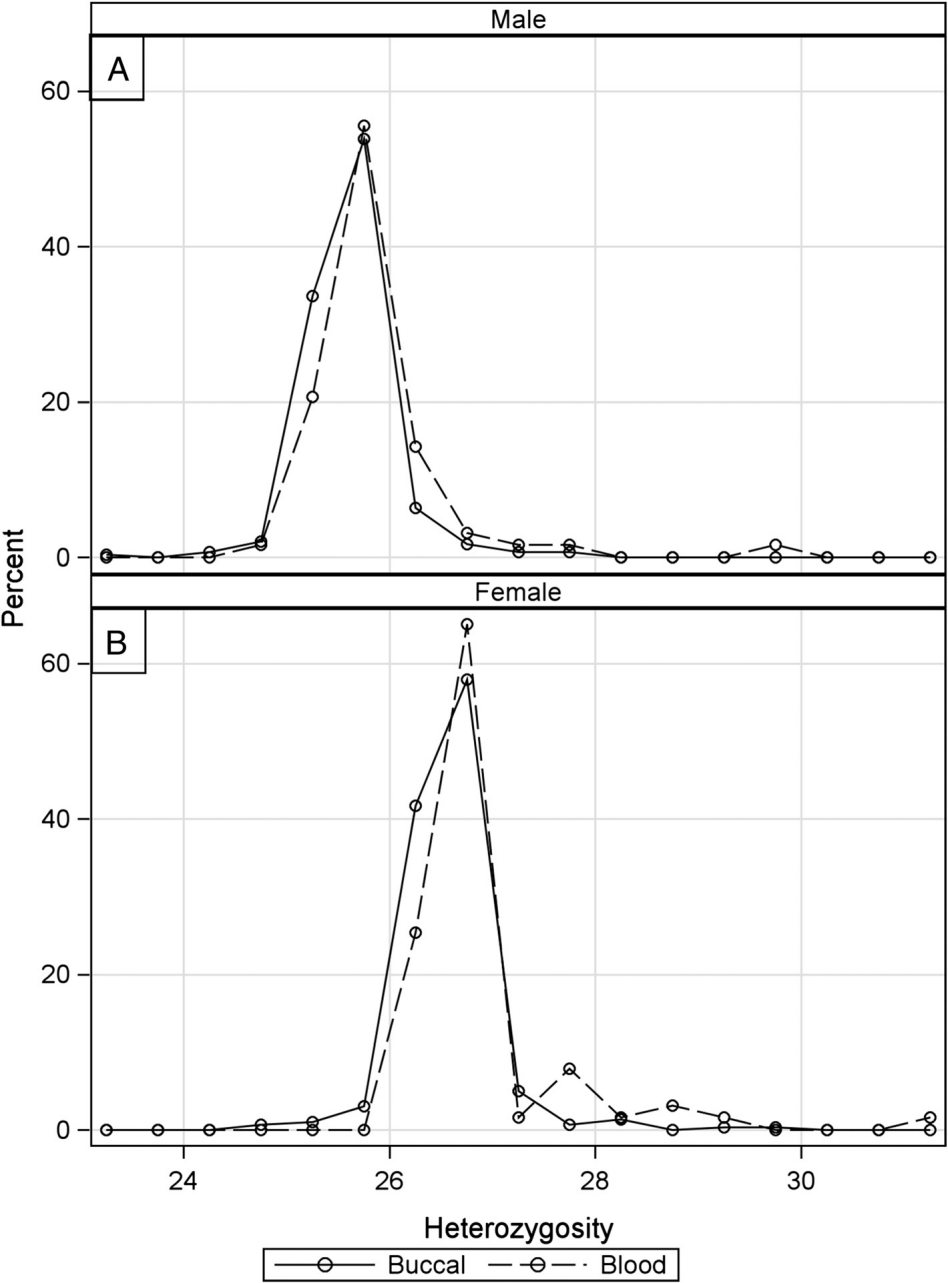
**Heterozygosity rates by sample type and sex.** Smoothed spline of heterozygosity rates calculated across all chromosomes, by sample type and sex for **A**) males and **B**) females. Heterozygosity rates for females are higher on average than males due to inclusion of X chromosome loci

Among samples that passed initial QC, sample call rates were modestly but significantly lower for buccal compared with blood DNA samples [Median (Interquartile Range, IQR): 99.1 (98.4, 99.5)% buccal vs. 99.4 (99.1, 99.8)% blood, p < 0.0001] (Figure 2). Heterozygosity rates were slightly but significantly lower in buccal samples for both male [Median (IQR): 25.6 (25.4, 25.8)% buccal vs. 25.7 (25.5, 25.9)% blood, p = 0.006; Figure 3A] and female samples [26.5 (26.4, 26.7)% buccal vs. 26.6 (26.5, 26.9)% blood, p = 0.001; Figure 3B].

**Figure 4:**
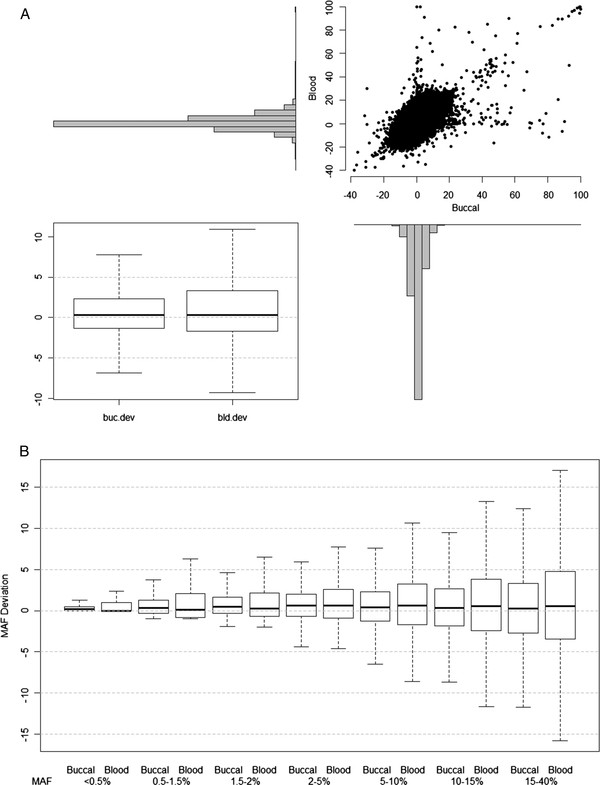
**MAF by sample type compared with CEU HapMap.** Panel **A**: Distributions and scatter plot of minor allele frequency (MAF) deviations from CEU HapMap reference by locus and sample type. Box plot represents overall deviation from the HapMap by sample type. Horizontal line represents median deviation, top and bottom box boundaries delineate the 25^th^ and 75^th^ percentiles (IQR) and whiskers extend to 1.5 times the IQR above and below the box. Panel **B**: MAF deviations by sample type, clustered by HapMap MAF categories. Box plots are constructed as in Panel A.

**Figure 5:**
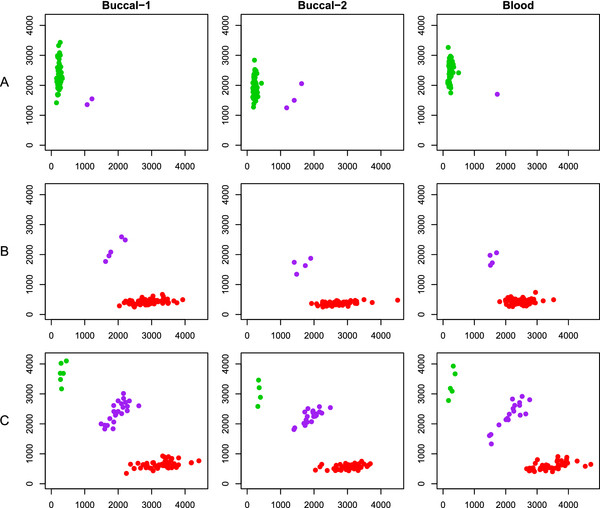
**Cluster plots by MAF and sample type.** Three SNPs meeting Hardy-Weinberg expectations (p > 0.24) were randomly selected for visual presentation, representing differing MAF classes. **A**) rs16933208 (MAF = 0.98%); **B**) rs12598047 (MAF = 2.88%); **C**) rs11803463 (MAF = 21.6%). Buccal-1 and Buccal-2 columns present clusters from two different plates of buccal samples, while the Blood column presents clusters from a plate of blood samples.

Of the 906,600 SNPs on the Affymetrix 6.0 chip, 755,837 (83%) had a MAF <40%. Overall, MAF for both buccal and blood samples were similar to estimates from CEU HapMap standards. Figure 4A shows the correspondence between buccal and blood sample deviations from CEU HapMap MAF. Overall, median MAF did not differ from HapMap for both blood and buccal samples (e.g., median absolute difference = 0), but blood samples had higher variability (IQR = −2% to +3% in blood, -1% to +2% in buccal). Figure 4B provides the deviation of our samples from the CEU MAF by sample type and MAF category. For all MAF categories, the median deviation from the CEU was between 0 and 1%, with interquartile ranges extending as far as 5% different from CEU. Typically, blood samples demonstrated slightly greater differences from CEU than buccal samples. Despite IQRs that included no difference, all comparisons of MAF overall or by MAF group were significant at p < 0.0001 due to very large numbers of loci included in estimates (>17,000 SNPs included in each MAF group). Visual review of cluster plots did not reveal any systematic difference between sample types for any MAF category (Figure 5).

## Discussion

The results of this study demonstrate that applying a minimum threshold for the ratio of double-stranded to total DNA quantity can improve the likelihood of successful genotyping for buccal DNA samples. Furthermore, applying this minimal level of quality control results in buccal genotyping results that are only slightly lower than the gold standard of blood DNA. This study also suggests specific additional considerations that may further improve results from buccal samples. Overall, we have shown that buccal samples not only can be successfully genotyped, but also provide high quality analytic data suitable for large-scale or high-throughput genetic studies.

The major finding of this study is that it is important to monitor ds/total DNA ratio to identify buccal samples at high likelihood of failure. In this study, low ds/total DNA ratio was a significant independent predictor of pre-genotyping failure, assuming acceptable 260/280 and 260/230 ratios. In this study, buccal samples drawn more recently were also more likely to fail, contrary to expectations, but time since sample collection did not alter the effects of ds/total DNA ratio in our analysis. Reasons for this finding are unclear, but may be driven statistically by a gap of over a year in which recruitment was dormant (e.g., artificially making the differences between the medians larger), as study protocols and staffing were consistent throughout the study. These results, however, suggest that longer cryogenic storage times do not negatively impact buccal DNA quality. The threshold of 34% ds/total DNA was selected to provide good discriminative value and low failure rate, as samples not meeting this threshold were 18 times more likely to fail than those meeting it. Application of this QC standard thus prevented the unnecessary running of samples on expensive chips if their likelihood of failure was significant, while retaining maximal sample size. This threshold may not be universally applicable to all studies depending on measurement of DNA concentration and laboratory techniques, but should be considered for studies conducting high-throughput genotyping using buccal-derived DNA.

In this large study, buccal samples had similar overall pre-genotyping failure rate, but lower cQC and lower DM call rates than blood samples, suggesting a shift toward lower overall quality. The similarity in overall failure rate may be somewhat optimistic, as buccal sample selection in the last 70% of this study was intentionally shifted toward those most likely to pass QC (e.g., ≥34% ds/total DNA); extracted samples below this threshold were not included in genotyping runs after the 3^rd^ plate. The current analysis suggests that buccal samples may require higher QC thresholds to ensure that cQC and DM quality metrics are comparable to blood samples, particularly for the DM call rate, which was lower for both passed and failed buccal samples compared with the blood samples. There is precedent for subjecting different “populations” of samples or loci to different thresholds to improve performance, which has shown promise in previous situations [14]. However, establishing higher thresholds would result in the rejection of more buccal samples in the QC phase, limiting sample size.

Birdseed genotyping results from passed samples were comparable for buccal and blood samples. Although all results were statistically better among blood samples, the magnitude of the differences are frequently nominal. This is consistent with a previous analysis of paired buccal and blood samples in this study that revealed similar call rates and 98.8% concordance of genotyping calls between buccal and blood samples, indicating high fidelity of genotyping across sample type [11]. Median call rates were high for both sample types (>99%) and deviations from HapMap minor allele frequencies for Caucasian populations were similarly minor. Analysis of MAF deviations showed similar patterns, even for rare SNPs that are frequently ignored in genetic studies for concern over accurate genotype calling. However, all MAFs estimated for this study deviated positively from HapMap estimates, which may be due to regional differences in population compared with the CEU HapMap. The overestimation of MAF may also suggest the presence of missing call bias where no-calls are more likely in one genotype than another [14], although this effect appears similar for buccal and blood samples. Visual inspection of cluster plots revealed no obvious issues, with distinct clusters and minimal no-calls for both sample types, even for SNPs of low MAF. Furthermore, the median test revealed no batch effects on estimated MAF by locus across plates (data not shown). However, the average raw intensities for buccal samples were significantly lower than the blood samples (data not shown), which suggests that buccal samples and blood samples should be clustered separately for accurate genotyping.

These similarities between buccal and blood samples suggest that both sample types can be used in high-throughput genetic studies with only minor caveats. However, with the increasing use of “out of study” controls in large-scale genetic studies, these minor differences may be magnified. In particular, when cases and controls are derived from different sample types (e.g., cases are buccal DNA, while controls are blood DNA), differences in sample failure rates, call rates and MAF estimation between buccal and blood samples may result in spurious false-positive associations with disease status [14]. It is thus imperative that sample types match between cases and controls, or are at least represent a balance of sample types in both groups.

There are several important strengths of this study. A large number of samples were evaluated in the context of an active genome-wide association study, and processed by the same laboratory personnel. Plates were constructed to incorporate both cases and matched controls of the same sample type on the same plate, and collection of buccal samples was conducted by trained study nurses using standardized protocols. Despite these significant strengths, this study also has certain limitations. The present analysis does not include paired buccal and blood samples from the same individuals, so we cannot determine concordance in genotyping between sample types. However, our previous study in the same population suggested buccal DNA provide reasonable concordance with paired blood samples [11]. Also, identification of the ds/total DNA ratio threshold was conducted in the course of the genotyping, which changed the decision about which samples would be genotyped in the remainder of study. Thus, it was not possible to fully evaluate whether the 34% threshold is optimal, or whether a different threshold would have been better if all samples had been run blinded to ds/total DNA ratio.

## Conclusions

Overall, buccal samples did not demonstrate significantly worse QC failure rates than blood samples, although some metrics show worse performance. Following stringent QC, the quality of final genotyping resulting from buccal samples is somewhat lower, but compares favorably to blood. There is no evidence for differential loss of information or bias with respect to genotype calling from buccal vs. blood DNA samples. We recommend using caution, but not doubt, when conducting high-throughput genetic studies with buccal samples.

## Methods

The Genetic and Environmental Risk Factors for Hemorrhagic Stroke (GERFHS) study is a large case–control study of hemorrhagic stroke in the Cincinnati, Ohio region. To maximize participation and representativeness of the cohort, buccal cytobrush collection was performed on the majority of subjects. Buccal brushes for genetic analysis were collected between 1997 and 2005, blood samples were collected for some subjects from 2000 to 2005, and blood was collected on all subjects after 2005. Each study participant included in this analysis had either a buccal sample or a blood sample genotyped, but not both. The GERFHS study employed a matched case–control design, with matching on age (±5 years), sex, and race; controls were also selected to have the same sample type (buccal or blood DNA samples) as their matched case whenever possible. All individuals included in this analysis were non-Hispanic white.

Buccal brushes were collected on each participant using CYTO-PAK Cytosoft Brushes (Medical Packaging Corp., Camarillo, CA). Study research nurses were trained to collect buccal cytobrush samples in a standardized fashion, as previously described [11]. Blood samples were drawn by hospital nursing personnel using two 10 ml purple-top tubes with EDTA solution from subjects during their hospital stay. Controls were recruited from the community using random-digit dialing. This study protocol was approved by the Institutional Review Boards of the University of Cincinnati and all participating hospitals, and all subjects provided written informed consent for genetic testing.

### DNA extraction and genotyping

Both blood and buccal cell DNA were extracted using PureGene DNA extraction kits specific to sample type (Gentra Systems, Inc. Minneapolis, MN), according to manufacturer directions. Briefly, the extraction for buccal DNA was as follows: buccal brushes were cut and placed into a microfuge tube containing cell lysis solution and proteinase K, and incubated overnight at 55°C. After cooling, protein precipitation solution was added, vortexed and centrifuged. The supernatant was transferred to a tube containing isopropanol and glycogen solution, incubated at room temperature, and centrifuged. The DNA pellet was washed with 70% ethanol and air dried. TE buffer was added and incubated at 1 hour at 65°C prior to storage at −20°C.

The total concentration, 260/280, 260/230 ratios of genomic DNA for all samples were measured using a spectrophotometer (NanoDrop ND-1000, NanoDrop Technologies). Finally, double-stranded DNA (dsDNA) concentration was measured using the Quant-it dsDNA BR assay kit and Qubit fluorometer (Invitrogen). All DNA samples were normalized to 50 ng/μl dsDNA in reduced EDTA TE buffer. Samples that did not meet 260/280 ratio of at least 1.7 and 260/230 ratio of at least 1.0 were not included in genotyping.

Cases and their matched controls were arrayed on the same plates, and their genotypes called at the same time. Genotyping was performed on the Affymetrix GeneChip Scanner 3000 platform using Human SNP Array 6.0. The recommended protocol as described in the Affymetrix manual was followed. Five μl (250 ng) of dsDNA was digested with *Sty I* and ligated to Sty I adapters using T4 DNA ligase. Another 5 μl (250 ng) of dsDNA was digested with *NspI* and ligated to Nsp I adapters using T4 DNA ligase. Two digested samples were then PCR amplified individually using TITANIUM DNA amplification kit (Clontech) on an ABI9700 machine. PCR products were pooled and purified using the Agencourt AMPure magnetic beads (Beckman Coulter) and 96-well filter plate (E&K Scientific) followed by fragmentation and labeling. Samples were then injected into cartridges, hybridized, washed, and stained. Mapping array images were obtained using the GeneChip Scanner 3000 with GCOS software. Image files were uploaded to the Cincinnati Children’s Hospital Medical Center (CCHMC) Genotyping Data Repository in 10 batches of ~96 samples per batch. Samples were determined to be initial QC failures if they failed to meet the default Birdseed (v. 1.12.0) QC thresholds of Contrast Quality Control (cQC) > 0.4, Dynamic Model (DM) call rate >83%, or were gender mismatches based on actual versus inferred gender. A subset of samples that failed initial QC were re-extracted and/or re-hybridized in an attempt to recover the sample; however, this rarely resulted in sample recovery. Samples that failed initial QC were excluded from full genotyping with Birdseed clustering. Birdseed genotyping was conducted using standard settings. Genotyping results were evaluated for evidence of batch effects using the median test, and results were not different by batch.

### Analysis design

All data analysis was conducted using SAS v.9.2 (SAS Institute, Cary, NC). Descriptive characteristics between participants with buccal versus blood samples were compared using parametric t-tests, non-parametric Wilcoxon rank sum, χ^2^ analyses, or Fisher’s Exact tests, as appropriate to the distribution of the variable.

The statistical evaluation of DNA performance was partitioned into two phases. The first phase was designed to evaluate the role of time since sample collection; relevant patient lifestyle (e.g., smoking or drinking alcohol or caffeinated beverages); and quantitative laboratory-based sample metrics for the ability to discriminate buccal DNA samples likely to fail pre-genotype calling quality control (QC) screening. Identification of independent predictors of sample failure was conducted in real-time (during the course of active genotyping) using data from the first three batches of samples (n = 270 buccal samples). Thresholds were established and then applied to the remaining samples run after that point. After the conclusion of all genotyping, analyses were conducted to compare QC metrics between buccal and blood samples and to calculate failure rates based on the previously established thresholds. This analysis sample set included results from both successful and failed buccal and blood samples, limited to one result per unique individual (n = 850) to avoid inflation of sample failure due to failed repeat hybridizations for some samples.

For this first phase of analysis, the dependent variable was success or failure of buccal DNA to pass pre-genotyping QC metrics. Independent variables for the first phase included the time between sample collection and genotyping; relevant patient lifestyle variables (current cigarette smoking, current cigar smoking, frequency of alcohol use, average daily intake of caffeinated coffee, tea or soda); and the quantity (total DNA concentration, dsDNA concentration, ds/total DNA ratio) and quality (260/280 and 260/230 ratios) of extracted DNA. Each variable was examined for outliers, and variables with excessive skewness or kurtosis (<−1 or >1) were natural log transformed to improve normality; both total DNA and dsDNA concentration were thus analyzed in log-transformed units. Because of differences in measurement method and sensitivity between the Nanodrop and Qubit DNA quantitation techniques, ds/total DNA ratios occasionally exceeded 1.0. Buccal DNA sample characteristics between samples that passed initial QC versus those that failed were compared using Wilcoxon rank sum statistics (for continuous variables) or Fisher’s Exact Test (for categorical variables). Logistic regression was used to construct receiver operator characteristic (ROC) curves and establish thresholds of significant variables to distinguish initial QC success from failure.

The second phase of analysis was designed to test the performance of buccal versus blood DNA samples that passed initial QC. Metrics for this analysis included sample call rate, minor allele frequency (MAF) comparisons between buccal and blood samples in reference to Caucasian (CEU) HapMap samples, and average heterozygosity rates. Heterozygosity rates were calculated separately by sex across all chromosomes. Call rates and heterozygosity rates between buccal and blood samples were compared using Wilcoxon Signed Rank tests.

Low MAF can influence the performance of the clustering algorithms for calling genotypes. Absolute differences between buccal or blood MAF and the CEU reference were analyzed using Wilcoxon Signed Rank tests. In addition, we explored the effect of specific MAF categories (MAF < 0.5%, 0.5%–1.5%, 1.5%–2%, 2%–5%, 5%–10%, 10%–15% and 15%–40%) on the deviation of buccal or blood sample MAF from the CEU HapMap standard. MAF categories were established based on the CEU HapMap reference, with MAF >40% excluded from analysis to minimize the likelihood that the minor allele in HapMap would be the major allele in our population.

## Abbreviations

CCHMC, Cincinnati Children’s Hospital Medical Center; CEU, HapMap population of Utah residents with northern and western European ancestry collected by the Centre d'Etude du Polymorphisme Humain (CEPH); cQC, Contrast quality control; DM, Dynamic model; dsDNA, Double-stranded DNA; GERFHS, Genetic and Environmental Risk Factors for Hemorrhagic Stroke; MAF, Minor allele frequency; QC, Quality control; ROC, Receiver operator characteristic.

## Competing interests

None of the authors reports any competing interests relative to the work presented in this manuscript.

## Authors’ contributions

JGW and LJM conceived of and designed the analysis, assessed genotyping quality control, conducted and oversaw statistical analysis and drafted the manuscript. LD conducted statistical analysis. WMB, TDH and CDL provided input to the study design and analysis, and provided critical review of the manuscript. CJM provided data management and phenotype identification. MH collected buccal swab samples and coordinated subject recruitment. GS, SRI and HC conducted all genotyping and initial quality control processing. RD oversaw the genotyping process and DW participated in the design of the sub-study and provided critical review of the manuscript. All authors read and approved the final manuscript.

## References

[B1] Hansen TV Simonsen MK Nielsen FC Hundrup YA Collection of blood, saliva, and buccal cell samples in a pilot study on the Danish nurse cohort: comparison of the response rate and quality of genomic DNA Cancer Epidemiol Biomarkers Prev 2007 16 10 2072 2076 10.1158/1055-9965.EPI-07-0611 17932355

[B2] Bahlo M Stankovich J Danoy P Hickey PF Taylor BV Browning SR Brown MA Rubio JP Saliva-derived DNA performs well in large-scale, high-density single-nucleotide polymorphism microarray studies Cancer Epidemiol Biomarkers Prev 2010 19 3 794 798 10.1158/1055-9965.EPI-09-0812 20200434

[B3] Rogers NL Cole SA Lan HC Crossa A Demerath EW New saliva DNA collection method compared to buccal cell collection techniques for epidemiological studies Am J Hum Biol 2007 19 3 319 326 10.1002/ajhb.20586 17421001 PMC2797479

[B4] Mulot C Stucker I Clavel J Beaune P Loriot MA Collection of human genomic DNA from buccal cells for genetics studies: comparison between cytobrush, mouthwash, and treated card J Biomed Biotechnol 2005 2005 3 291 296 10.1155/JBB.2005.291 16192688 PMC1224694

[B5] Steinberg K Beck J Nickerson D Garcia-Closas M Gallagher M Caggana M Reid Y Cosentino M Ji J Johnson D Hayes RB Earley M Lorey F Hannon H Khoury MJ Sampson E DNA banking for epidemiologic studies: a review of current practices Epidemiology 2002 13 3 246 254 10.1097/00001648-200205000-00003 11964924

[B6] Garcia-Closas M Moore LE Rabkin CS Franklin T Struewing J Ginzinger D Alguacil J Rothman N Quantitation of DNA in buccal cell samples collected in epidemiological studies Biomarkers 2006 11 5 472 479 10.1080/13547500600733820 16966163

[B7] King IB Satia-Abouta J Thornquist MD Bigler J Patterson RE Kristal AR Shattuck AL Potter JD White E Buccal cell DNA yield, quality, and collection costs: comparison of methods for large-scale studies Cancer Epidemiol Biomarkers Prev 2002 11 10 Pt 1 1130 1133 12376522

[B8] Moore LE Bergen AW Haque KA Qi Y Castle P Chanock SJ Egan K Newcomb P Titus-Ernstoff L Alguacil J Rothman N Garcia-Closas M Whole genome amplification of buccal cytobrush DNA collected for molecular epidemiology studies Biomarkers 2007 12 3 303 312 10.1080/13547500601162011 17453743

[B9] Sigurdson AJ Ha M Cosentino M Franklin T Haque KA Qi Y Glaser C Reid Y Vaught JB Bergen AW Long-term storage and recovery of buccal cell DNA from treated cards Cancer Epidemiol Biomarkers Prev 2006 15 2 385 388 10.1158/1055-9965.EPI-05-0662 16492933

[B10] Feigelson HS Rodriguez C Robertson AS Jacobs EJ Calle EE Reid YA Thun MJ Determinants of DNA yield and quality from buccal cell samples collected with mouthwash Cancer Epidemiol Biomarkers Prev 2001 10 9 1005 1008 11535555

[B11] Woo JG Sun G Haverbusch M Indugula S Martin LJ Broderick JP Deka R Woo D Quality assessment of buccal versus blood genomic DNA using the Affymetrix 500 K GeneChip BMC Genet 2007 8 79 17996058 10.1186/1471-2156-8-79 PMC2174507

[B12] Feigelson HS Rodriguez C Welch R Hutchinson A Shao W Jacobs K Diver WR Calle EE Thun MJ Hunter DJ Thomas G Chanock SJ Successful genome-wide scan in paired blood and buccal samples Cancer Epidemiol Biomarkers Prev 2007 16 5 1023 1025 10.1158/1055-9965.EPI-06-0859 17507632

[B13] Rincon G Tengvall K Belanger JM Lagoutte L Medrano JF Andre C Thomas A Lawley CT Hansen MS Lindblad-Toh K Oberbauer AM Comparison of buccal and blood-derived canine DNA, either native or whole genome amplified, for array-based genome-wide association studies BMC Res Notes 2011 4 226 10.1186/1756-0500-4-226 21718521 PMC3145587

[B14] Fu W Wang Y Li R Lin R Jin L Missing call bias in high-throughput genotyping BMC Genomics 2009 10 106 10.1186/1471-2164-10-106 19284636 PMC2670840

